# No relationship between chronotype and timing of breeding when variation in daily activity patterns across the breeding season is taken into account

**DOI:** 10.1002/ece3.9353

**Published:** 2022-09-20

**Authors:** Marjolein Meijdam, Wendt Müller, Bert Thys, Marcel Eens

**Affiliations:** ^1^ Department of Biology, Behavioural Ecology and Ecophysiology Group University of Antwerp Wilrijk Belgium

**Keywords:** active daylength, chronotype, circadian rhythm, clutch initiation date, emergence time, *Parus major*

## Abstract

There is increasing evidence that individuals are consistent in the timing of their daily activities, and that individual variation in temporal behavior is related to the timing of reproduction. However, it remains unclear whether observed patterns relate to the timing of the onset of activity or whether an early onset of activity extends the time that is available for foraging. This may then again facilitate reproduction. Furthermore, the timing of activity onset and offset may vary across the breeding season, which may complicate studying the above‐mentioned relationships. Here, we examined in a wild population of great tits (*Parus major*) whether an early clutch initiation date may be related to an early onset of activity and/or to longer active daylengths. We also investigated how these parameters are affected by the date of measurement. To test these hypotheses, we measured emergence and entry time from/into the nest box as proxies for activity onset and offset in females during the egg laying phase. We then determined active daylength. Both emergence time and active daylength were related to clutch initiation date. However, a more detailed analysis showed that the timing of activities with respect to sunrise and sunset varied throughout the breeding season both within and among individuals. The observed positive relationships are hence potentially statistical artifacts. After methodologically correcting for this date effect, by using data from the pre‐egg laying phase, where all individuals were measured on the same days, neither of the relationships remained significant. Taking methodological pitfalls and temporal variation into account may hence be crucial for understanding the significance of chronotypes.

## Graphical abstract

Initially, both the timing of activity onset and active daylength seemed to be related to clutch initiation date in female great tits. However, we found significant changes in the timing of activity onset and active daylength over the breeding season and the initial relationships are hence confounded by date of measurement. Taking methodological pitfalls and seasonal variation into account may be crucial for understanding the significance of chronotypes.

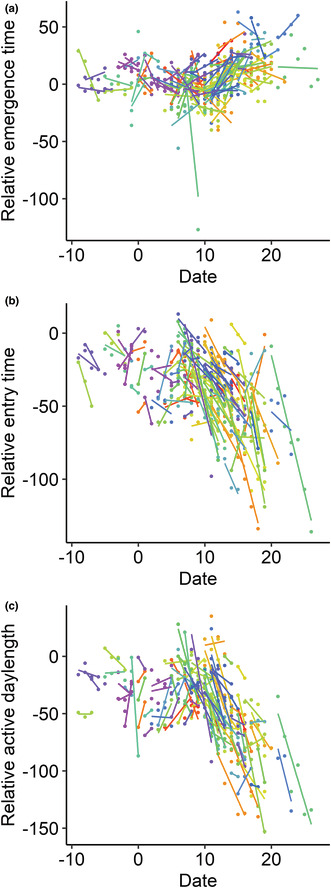

## 1 INTRODUCTION

Circadian rhythms occur on a diel (24 h) time scale and are ubiquitous in all living organisms. They are endogenously orchestrated by the biological clock, but entrained by the light–dark cycle, so that they match the 24 h daylength (Pittendrigh, 1993). However, the free‐running period length (*τ*), which represents the amount of time it takes the endogenous clock to repeat itself in the absence of environmental cues, often differs slightly from 24 h and it intriguingly varies among individuals too (Helm & Visser, 2010). This individual variation in the functioning of the biological clock becomes visible at the phenotypic level as consistent among‐individual variation in the timing of activities. The early or late timing of events is referred to as “chronotype.” It typically captures the timing when an individual starts with its activity in the morning and when it becomes inactive in the evening. In humans, variation in the preferred timing of activities is referred to as “morningness” and “eveningness” (Arrona‐Palacios et al., 2020). Variation in the timing of activity patterns have been found in a variety of other taxa, including mammals and birds, both in laboratory settings and free‐living populations (e.g., Labyak et al., 1997; Lehmann et al., 2012; Refinetti et al., 2016; Steinmeyer et al., 2010). Thus, it is commonly accepted that individuals consistently differ from each other in the timing of their activity patterns.

Understanding how this individual variation in chronotypes is maintained in natural populations is of outermost relevance, but knowledge about the evolution and adaptive significance of chronotypes in natural ecosystems is still scarce (Dominoni et al., 2017; Helm et al., 2017). However, recently there is an increased interest in this topic. Furthermore, while existing studies are often laboratory‐based, where testing functional consequences or even fitness consequences is difficult (Van der Veen et al., 2017), studies on chronotypes are now taken into the wild. Here, it can be expected that chronotypes are under both sexual and natural selection, as chronotypes may influence the timing of the expression of certain traits (Hau et al., 2017). For example, dawn song in male birds should be timed precisely to the presence of (receptive) females (Hau et al., 2017), while timing might also play an important role for minimizing predation risk and maximizing foraging efficiency (DeCoursey et al., 2000; Helm et al., 2017).

Still, empirical evidence on the fitness consequences of chronotypes is mixed. Both male and female birds that engaged in extra pair copulations, which particularly occur at dawn, had earlier chronotypes than other birds (Halfwerk et al., 2011; Poesel et al., 2006), but this could not be confirmed in a later study (Schlicht et al., 2014). Maury et al. (2020) and Steinmeyer et al. (2013) found that clutch size and number of fledglings were independent from temporal phenotype in females, but Graham et al. (2017) reported that females which had an earlier onset of activity in the morning had earlier clutch initiation dates. The latter is commonly assumed to be a fitness measure, as earlier hatched chicks have higher recruitment rates (e.g., Verboven & Visser, 1998). This suggests that the timing of reproduction rather than the reproductive investment might vary with chronotype.

However, if early rising females have a similar timing for the offset of activity as late rising females, this would lengthen their active day (i.e., the time they spend outside the nest box) and the time they can, for example, spend on foraging. Early rising, and thus increasing active daylength, would then allow individuals to make more efficiently use of the limited resources at the beginning of the breeding season, as they would have more time available. The active daylength can be further increased by delaying the cessation time, as has recently been reported for female European starlings (*Sturnus vulgaris*), where individuals with an early onset of activity had later cessation times than females which had a late onset of activity (Maury et al., 2020). Also in blue tits (*Cyanistes caeruleus*), substantial variation among individuals has been shown for active daylength, so that a distinction between long‐ and short‐sleeping individuals could be made (Steinmeyer et al., 2010). This altogether implies that a relationship between activity onset in the morning and clutch initiation date may not only depend on the timing of daily activity but could also be the result of an increase in active daylength in early rising individuals.

Furthermore, a concern that has potentially not sufficiently been taken into account in previous studies on the fitness consequences of the daily timing of activity is the contribution of temporal variation across the breeding season as underlying driver of such relationships between fitness and timing of activity. Emergence time, entry time, and therewith active daylength, which are key parameters when studying individual variation in temporal behavior, vary throughout the year (Schlicht & Kempenaers, 2020; Steinmeyer et al., 2010; Stuber et al., 2015), even after correcting for the seasonal changes in the timing of sunrise and sunset. This suggests that the significance of sunrise and sunset for determining activity patterns may differ across the year or with date of measurement both within and among individuals. The date of measurement may thus be a confounding factor when analyzing relationships between the activity parameters and fitness estimates such as clutch initiation date, which are temporal parameters in itself.

Here, we study the relationships between activity patterns at the onset of reproduction and clutch initiation date (Graham et al., 2017), as measured by regular nest checks in a nest box breeding population of great tits. First, we investigate whether individual variation in activity patterns is consistent (i.e., repeatable) within and across periods (pre‐egg laying and egg laying) in the breeding cycle. Then, we investigate whether the daily timing of onset of activity in the morning is related to the seasonal timing of onset of reproduction, that is, start of egg laying. By considering both onset (here: emergence time from the nest box) and offset (here: nest box entry time in the evening) of daily activity, we also investigate the hypothesis that earlier rising females have longer active daylengths (i.e., advanced onset but not advanced offset), which allows them to accumulate the relevant resources earlier in the breeding season, so that they can start reproduction earlier in the season. Finally, we investigate whether the above described relationships may be affected by variation in the daily timing of activity across the breeding season.

## 2 MATERIALS AND METHODS

### 2.1 Population

This study was carried out in a suburban nest box population of great tits, located in Wilrijk (Antwerp), Belgium (51°09′46.1”N, 4°24′13.3″ E) during the breeding season (March–June) of 2020 (Raap et al., 2016; Rivera‐Gutierrez et al., 2012; Van Duyse et al., 2005). About 170 nest boxes, suitable for great tits, are placed in trees at a height of about 2 m. All individuals that had been captured during previous breeding seasons or during roosting in winter were equipped with a ring containing a PIT‐tag (passive integrated transponder; EM4102, 125 KHz, Eccel Technology Ltd) and a unique combination of color rings, enabling individual recognition. The nest boxes were checked every few days for nest building, egg laying, and incubation. In our population, great tits can have up to two broods per year, but this study only contains data of first breeding attempts.

### 2.2 Emergence and entry times

To determine the time at which females leave the nest box in the morning (emergence time) and enter in the evening (entry time), we used SongMeters (SongMeter™ SM2+; Wildlife Acoustics, Inc) and radio‐frequency identification (RFID) loggers (EM4102 data logger, Eccel Technology Ltd). RFID loggers consist of two antennas, which were placed around the opening of the nest box, one on the inside, the other on the outside. When a PIT‐tagged individual passes through the antennas, the RFID logger registers the unique PIT‐tag number and the time of passing (for more details, see Iserbyt et al., 2018). The reader sample interval was set to 250 ms and the sleep mode between 10:00 p.m. and 03:00 a.m. As not all individuals in the population were equipped with PIT‐tags, we also used SongMeters to determine the emergence and entry times. SongMeters have two microphones to record sounds both inside and outside the nest box. Both microphones produce sonograms. Before the clock changed to summer time, sound was recorded in the morning from 04:00 a.m. to 08:00 a.m. CET and in the evening from 05:30 p.m. to 08:30 p.m. CET. After the clock changed to summertime, we recorded sound from 03:00 a.m. to 08:00 a.m. CET in the morning and in the evening from 05:30 p.m. to 09:00 p.m. CET. Morning emergence time and evening entry time could be determined by the sound of the female's claws on the nest box (microphone inside) and the sound of her wings when taking off (microphone inside and outside; Halfwerk et al., 2011). Furthermore, a specific sound caused by a change in air pressure can be heard when the female passes the opening of the nest box. Data recorded by SongMeters were analyzed using Avisoft SASLab Pro 5.2.14 (Specht, 2002).

Emergence and entry times were measured during the egg laying phase (i.e., after the first egg was laid and before incubation started) and for a subset of individuals also during the pre‐egg laying phase (i.e., when nest building was completed and before the first egg was laid; see below). As individuals shift the timing of activity substantially between the different stages of breeding (Schlicht & Kempenaers, 2020), the physiological state should not differ between individuals when measuring activity patterns. During the pre‐egg laying phase, all individuals should thus be measured once nest building is completed. However, not all females sleep in the nest box during this phase, and many females finish nest building only the day before egg laying starts. This does not allow obtaining large sample sizes during the pre‐egg laying phase. During the egg laying phase however, all females sleep in the nest box and measuring all individuals in the same physiological state is relatively easy. As timing of activity is thought to be consistent we expected that individuals with relatively early timing during the egg laying phase would also be early during the pre‐egg laying phase. We showed that emergence time is repeatable on the long term (i.e., across years) in female great tits in our population (Meijdam et al., 2022). Therefore, we decided to measure emergence and entry times mainly during the egg laying phase.

We used a combination of both SongMeters and RFID loggers. Emergence times were measured 88 times with both SongMeter and RFID logger. Twenty‐seven percent of the measurements by RFID loggers did not correspond with the SongMeter. Visual validation of our RFID loggers was performed in previous years both in blue tits and great tits. In blue tits, in a dataset of 242 parental visits (*N* = 10 nests), 86.8% of all entries and 43.8% of all departures were registered (Iserbyt et al., 2018). In great tits, the correlation between feeding rates of females measured with RFID loggers and cameras was 0.78 (Thys et al., 2021; note: when feeding chicks females both enter and depart from the nest box so there are 2 chances to be registered). Thus, even though the speed when passing the RFID logger is much higher during chick rearing when compared with leaving the box after awakening, and is also faster in blue tits, there is still a change that the entry or emergence time into/from the nest box will be missed by our RFID loggers. In almost all instances in which the SongMeter data did not correspond to the RFID logger data, the RFID logger showed later emergence times and earlier entry times than the SongMeter. Therefore, SongMeter data are likely more accurate and it is highly likely that the RFID loggers missed the first emergence and last entry from/into the nest box. Unfortunately, we do not have data to visually validate the data collected by SongMeters. However, determining emergence and entry times using SongMeters is straight forward (see Figure S1) and has successfully been used in previous papers (e.g., Halfwerk et al., 2011).

For these reasons, we decided to use only data from SongMeters if both SongMeter and RFID logger data were available. If only RFID logger data were available (*n*_observations_ = 58 on 30 females), only measurements that fell within the range of emergence times measured by the SongMeters were included in the dataset (127 min before sunrise up to 63 min after sunrise; this resulted in the removal of 16 datapoints). For entry times, the error was 12% on 82 measurements, so here, we used the same procedure as for emergence times (an overview of the sample sizes after the data removal criterion was applied is presented in Table 1. A comprehensive overview of the number of birds sampled per day is presented in Table S1). Completely excluding the RFID data from the analyses did not change the outcome or interpretation (these results will not be further discussed).

**TABLE 1:** Sample sizes of emergence time, entry time and active daylength during the pre‐egg laying phase and the egg laying phase.

Phase	Variable	Number of females	Number of measurements							
			Mean per female	Repeats per female						
				1	2	3	4	5	6	7
Pre‐egg laying	Emergence time	23	2.96		1	22	0			
	Entry time	24	3.04		3	17	4			
	Active daylength	22	2.95		1	21	0			
Egg laying	Emergence time	121	3.84	1	5	49	27	36	2	1
	Entry time	116	3.54	3	23	30	35	19	5	1
	Active daylength	114	2.98	2	45	26	36	4	1	0

For both SongMeter and RFID data, we determined emergence times relative to sunrise (negative = before sunrise, positive = after sunrise) and entry times relative to sunset (negative = before sunset, positive = after sunset). We also determined the relative active daylength (negative = shorter active period than the period between sunrise and sunset, positive = longer active period than the period between sunrise and sunset). Hereafter, emergence time, entry time, and active daylength always concern relative times, unless it is specifically made clear that they concern absolute times. Temperature data was retrieved via: https://www.wunderground.com/history/daily/be/antwerp.

In models containing emergence time, we used the temperature (*T*°) at sunrise, and in models containing entry time, we used *T*° at sunset, and in models containing active daylength, we used the maximum daily *T*° on the day of measurement.

### 2.3 Statistical analysis

All statistical analysis were performed in R 4.0.2 (R Core Team, 2013). We used the “rptR” package (Stoffel et al., 2017) to calculate repeatabilities, which uses parametric bootstrapping to quantify confidence intervals, and likelihood ratio testing to determine statistical significance. Statistical significance of fixed effects for each linear mixed model was obtained with stepwise backwards elimination using lmerTest (Kuznetsova et al., 2017). For all statistical tests, the significance level was set at *α* = 0.05.

To test if an individual's average entry time depended on its average emergence time, both measured during the egg laying phase, a linear model was used. Second, we used two separate linear models to test whether clutch initiation date depended on the individual's average emergence time or its average active daylength. Clutch initiation dates ranged from March 22 up to April 20 (=30 days). In both models, female age (years) was included as fixed effect.

Although we used relative values for emergence and entry time to account for changes in the onset of dawn and dusk across the breeding season, a visual inspection of the data revealed that there could still be temporal variation in both parameters. To explore these patterns, we modeled variation in activity parameters in relation to the date of measurement, using random regression analyses (Dingemanse et al., 2010; Nussey et al., 2007). Three identical models were run for emergence time, entry time, and active daylength. The models included the average date (starting as a count from April 1) on which an individual was measured (=among‐individual effect), the deviation from the average date (=within‐individual effect; Van de Pol & Wright, 2009), their interaction and age of the female as fixed effects. The among‐individual effect allows to test whether females, on average (population‐level), differ in activity patterns when observed on different dates. The within‐individual effect allows to test whether females, on the population level, plastically adjust their activity as the date progresses. The interaction allows to test whether plasticity depends on mean date of testing. As temperature is known to affect the activity patterns in great tits (Lehmann et al., 2012; Stuber et al., 2015), we also included the temperature as described above. Random intercepts (=chronotype; i.e., do individuals differ from each other in average activity patterns?) were included for female ID and random slopes on the level of the deviation from the average date (=individual plasticity in activity patterns in response to date; i.e., do individuals differ from each other in plasticity?) were included for female ID as well. Stepwise backwards elimination of non‐significant terms was performed to obtain the minimum adequate model (MAM). Likelihood ratio tests were used to determine significance of random effects (i.e., individual intercept and slope). Adjusted repeatabilities for emergence time, entry time, and active daylength during the egg laying phase were calculated from these MAMs as the variance explained by female ID relative to the total variance.

As we suspected that the variation in emergence time, entry time, and active daylength across the breeding season may have confounded the relationships between clutch initiation date and emergence time/active daylength, we decided to use additional data that we had collected during the pre‐egg laying phase. During this phase, we placed SongMeters on 25 nest boxes with nests that were completed, but with no eggs yet. For these 25 females, we measured emergence and entry times between March 26 and March 30. Temporal variation was thus very limited. We used linear mixed models to test whether emergence time, entry time, and active daylength were affected by the date of measurement, the number of days prior to clutch initiation, the temperature, and the female's age (in years since birth, with age = 0 is year of birth; for the results, see Figure S2 and Table S2). In all models, random intercepts were included for female identity (ID). After excluding non‐significant fixed effects, we calculated the adjusted repeatability.

We performed similar analyses as before to determine the relationships between emergence time/active daylength measured during the pre‐egg laying phase and clutch initiation date. Here, clutch initiation dates ranged from March 29 up to April 15 (=18 days).

Additionally, using a separate model on the subset of females measured during both the pre‐egg laying and the egg laying phase, we estimated the between‐period repeatability of emergence time, entry time, and active daylength (*n*_emergence time_ = 23, *n*_entry time_ = 24, *n*_active daylength_ = 21). We included the reproductive phase as a two‐level factor (i.e., pre‐egg laying vs. egg laying) and the measurement interval (i.e., the number of days between the average measurement date during the pre‐egg laying phase and the egg laying phase; mean = 8.3, min. = 3, max = 18.5) as a continuous covariate. Non‐significant fixed effects were removed from the models. Both female ID and the unique combination of period and female ID were included as random effects, thereby specifically allowing to estimate the between‐period repeatability, following Araya‐Ajoy et al. (2015). That is, the adjusted between‐period repeatability was calculated from this model as the variance explained by the individual relative to the total variance.

### 2.4 Ethical note

This study was approved by the ethical committee of the University of Antwerp (ID numbers: 2016–87 and 2018–50) and was performed in accordance with Belgian and Flemish laws regarding animal welfare, adhered to the ASAB/ABS guidelines for the use of animals in behavioral research and teaching, and complies with ARRIVE guidelines. The Royal Belgian Institute of Natural Sciences (KBIN) provided ringing licenses for all authors and technicians. Handling time was minimized as much as possible. All other methods described above are non‐invasive.

## 3 RESULTS

During the pre‐egg laying phase, emergence times ranged between 76 min before sunrise and 21 min after sunrise (Table 2). Entry times ranged from 63 minutes before sunset up to 10 minutes after sunset and active daylengths from 56 min shorter than the daylight period up to 46 min longer. During the egg laying phase, emergence times ranged from 127 min before sunrise up to 63 min after sunrise. Entry times ranged between 136 min before sunset and 13 min after sunset. The shortest active daylength we measured was 153 min shorter than the daylight period and the longest active day was 35 min longer than the daylight period.

**TABLE 2:** Summary of the measured values for emergence time, entry time and active daylength (in minutes relative to sunrise, sunset and the period between sunrise and sunset respectively) during the pre‐egg laying and egg laying phase.

Phase	Variable	Min.	Max.	Mean	SD
Pre‐egg laying	Emergence time	−76	21	−17.51	13.64
	Entry time	−63	10	−23.07	17.25
	Active daylength	−56	46	−6.25	21.92
Egg laying	Emergence time	−127	63	5.81	17.59
	Entry time	−136	13	−39.18	24.67
	Active daylength	−153	35	−45.35	32.51

### 3.1 Repeatability of daily activity patterns

Both during the pre‐egg laying phase and the egg laying phase, the adjusted repeatability was significant for emergence time, entry time, and active daylength (Table 3). In contrast, between‐period repeatabilities for emergence time (*R* [95% CI] = 0.09 [0, 0.30]), entry time (*R* = 0.20 [0, 0.42]), and active daylength were not significant (*R* = 0 [0, 0]).

**TABLE 3:** Adjusted repeatability for emergence time, entry time, and active daylength (in minutes relative to sunrise, sunset, and the period between sunrise and sunset respectively) during the pre‐egg laying and egg laying phase.

Phase	Variable	Adjusted repeatability
Pre‐egg laying	Emergence time	**0.39 [0.10, 0.62]**
	Entry time	**0.27 [0.018, 0.52]**
	Active daylength	**0.45 [0.17, 0.67]**
Egg laying	Emergence time	**0.54 [0.43, 0.63]**
	Entry time	**0.77 [0.71, 0.83]**
	Active daylength	**0.71 [0.63, 0.80]**

*Note*: All repeatabilities were calculated based on the MAM for the respective period and variable (for information on significant fixed effects see Table S2 and Table 4). 95% confidence intervals are shown between brackets. Estimates in bold are statistically significant (*p* < .05).

### 3.2 Clutch initiation date and daily activity patterns during the egg laying phase

**FIGURE 1:**
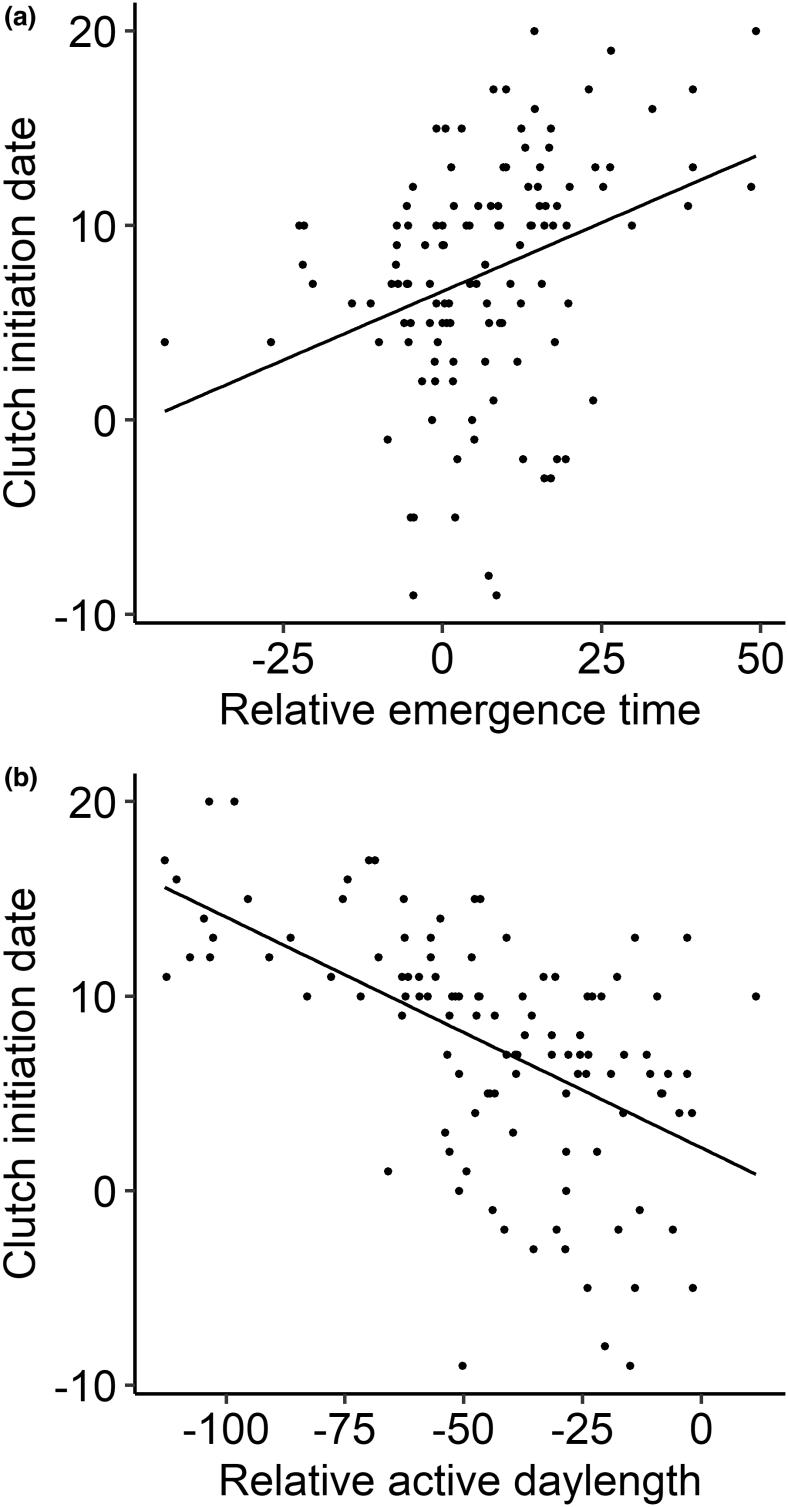
Average emergence times in minutes relative to sunrise (negative value = before sunrise) (a) and average active daylength in minutes relative to the period between sunrise and sunset (negative value = shorter active than the period between sunrise and sunset) (b) as measured during the egg laying phase both affected the clutch initiation date (starts as a count from April 1 (= 1)).

Females with an earlier emergence time during the egg laying phase ended their activities outside the nest box later during the day than females that showed a later onset of activity (*t* = −2.62, df = 112, *p* < .01). Emergence time was positively related to clutch initiation date (*t* = 3.85, df = 118, *p* < .001; Figure 1a). Individuals that started their activity early during the day laid their first egg earlier during the breeding season than individuals with late emergence times. In addition, active daylength was negatively related to clutch initiation date (*t* = −6.96, df = 111, *p* < .001; Figure 1b). Individuals that were longer active during the day laid their first egg earlier during the breeding season than individuals that were active for a shorter time period.

### 3.3 The influence of date on daily activity patterns

**FIGURE 2:**
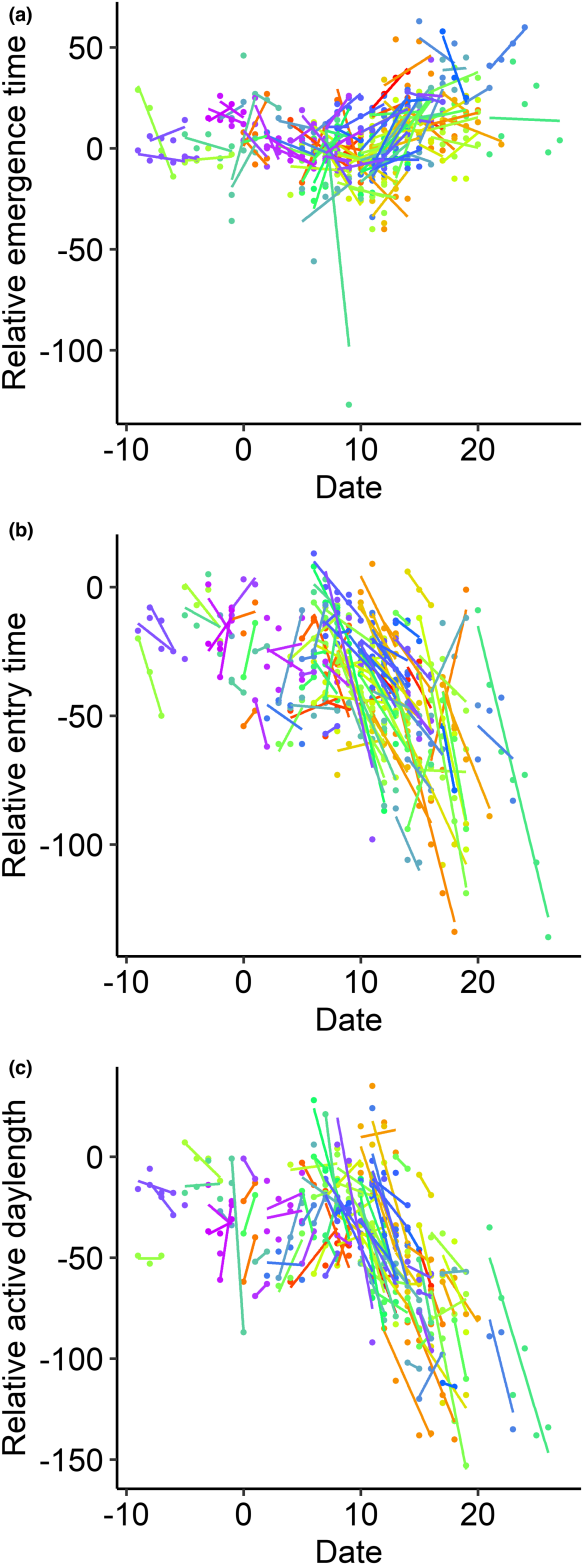
Activity patterns in female great tits are dependent on the date: (a) emergence times relative to sunrise, (b) entry times relative to sunset, and (c) active daylength in minutes relative to the period between sunrise and sunset. All individuals have separate regression lines (individuals can be distinguished by color). Date starts as a count from April 1 (= 1).

Date had an important influence on emergence time, entry time, and active daylength (Figure 2; Table 4). Early in the season individuals emerged close to sunrise (=0), while later in the season emergence times became later (=positive values; Figure 2a). Thus, date had a positive effect on emergence times (Average date effect is given in Table 4). This effect was partly driven by an among‐individual effect, but at the same time, emergence time became later on consecutive days within individuals (Date deviation effect in MAM: *t* = 3.23, df = 296.72, *p* < .01; Figure 2a). Furthermore, older females had earlier emergence times (Table 4). Temperature at sunrise did not affect emergence times. On average, entry time became earlier as the date progressed (Figure 2b) and active daylength shorter (Figure 2c; Table 4). Within individuals, these effects on entry time and active daylength became in both cases stronger toward the end of the breeding season, as indicated by the significant interaction between average date and date deviation (Table 4). Age did not have an effect on either entry time or active daylength. Active daylength was longer on warmer days (Table 4), but temperature at sunset did not affect entry time.

**TABLE 4:** Results from linear mixed effects models with random intercepts and slopes for testing the influence of date on emergence time, entry time and active daylength (in minutes relative to sunrise, sunset and the period between sunrise and sunset respectively) during the egg laying phase.

Dependent variable	Fixed effects	*β*	SE	*t*	df	*p*
Emergence time	Average date	**0.61**	**0.21**	**2.94**	**106.72**	**<.01**
	Date deviation	1.25	1.31	0.95	80.28	.34
	Age	**−2.98**	**1.29**	**−2.31**	**92.37**	**.02**
	*T*_sunrise_	−0.31	0.25	−1.25	350.26	.21
	Average date × date deviation	0.11	0.12	0.95	76.49	.56
	Random effects	*σ* ^2^		χ^2^	df	*p*
	ID_intercept_	**118.07**		**119.34**	**1**	**<.001**
	ID_slope_	14.33		3.79	2	.15
	Corr_intercepts‐slopes_	−0.03				
	Residual	93.14				
Entry time	Fixed effects	*β*	SE	*t*	df	*p*
	Average date	**−1.96**	**0.27**	**−7.19**	**99.32**	**<.001**
	Date deviation	0.66	2.45	0.27	111.04	.79
	Age	−1.79	1.62	−1.11	87.96	.27
	*T*_sunset_	0.14	0.17	0.81	293.66	.42
	Average date × date deviation	**−0.94**	**0.21**	**−4.48**	**99.28**	**<.001**
	Random effects	*σ* ^2^		*χ* ^2^	df	*p*
	ID_intercept_	**212.57**		**114.89**	**1**	**<.001**
	ID_slope_	**83.25**		**50.76**	**2**	**<.001**
	Corr_intercepts‐slopes_	0.32				
	Residual	87.23				
Active daylength	Fixed effects	*β*	SE	*t*	df	*p*
	Average date	**−2.71**	**0.34**	**−7.94**	**105.84**	**<.001**
	Date deviation	−0.54	3.07	−0.18	75.59	.86
	Age	0.34	2.11	0.16	87.68	.87
	*T*_max_	**0.76**	**0.26**	**2.91**	**262.75**	**<.01**
	Average date × date deviation	**−1.05**	**0.27**	**−3.87**	**73.51**	**<.001**
	Random effects	*σ* ^2^		*χ* ^2^	df	*p*
	ID_intercept_	**304.74**		**92.30**	**1**	**<.001**
	ID_slope_	**83.97**		**6.83**	**2**	**.03**
	Corr_intercepts‐slopes_	0.01				
	Residual	184.06				

*Note*: Estimates in bold are statistically significant (*p* < .05).

### 3.4 Clutch initiation date and activity patterns during the pre‐egg laying phase

During the pre‐egg laying phase entry times were not related to emergence times (a subset of females, *n* = 23, *t* = −0.44, df = 22, *p* = .66). Furthermore, during the pre‐egg laying phase, neither emergence time (*t* = −0.25, df = 3,18, *p* = .81), nor active daylength (*t* = 1.58, df = 2,19, *p* = .13) were related to clutch initiation date.

## 4 DISCUSSION

Our initial analysis supported the previously reported finding that female great tits with an early onset of activity start to reproduce earlier in the season (Graham et al., 2017). The data equally supported our hypothesis that the relationship between clutch initiation date and emergence time is driven by active daylength, that is, the time a female has available for foraging. However, when taking the date effect on the activity measures into account, by using data from the pre‐egg laying phase (where all individuals were measured on the same days), neither of these relationships remained significant. The consequences thereof for this and previous studies will be discussed below.

### 4.1 Repeatability

During the egg laying phase, repeatability of all activity measures was high, which suggests the existence of chronotypes in female great tits (Lehmann et al., 2012; Maury et al., 2020; Schlicht & Kempenaers, 2020). Also during the pre‐egg laying phase, activity measures were moderately and significantly repeatable. Contrary to our expectation, the between‐period repeatability (i.e., across the pre‐egg laying and egg laying phase) of emergence time, entry time, and active daylength was non‐significant. When studying the relationship between daily timing of activity and clutch initiation date, the timing of activity should thus preferentially be measured before egg laying starts. However, in our initial analyses and in previous studies on this relationship, the timing of activity was measured during later periods (Here: egg laying phase, Graham et al., 2017: incubation, Maury et al., 2020: incubation, Helm & Visser, 2010: autumn). The lack of repeatability between the different periods may be due to the small sample sizes during the pre‐egg laying phase. Especially for longer term repeatability small sample sizes can cause great imprecision in the estimate for among‐individual variation and low power to detect significance, which affects the repeatability estimate (Araya‐Ajoy et al., 2015). Therefore, it will be of interest to investigate long‐term (i.e., cross‐season and cross‐year) repeatability of activity patterns in more detail and with larger sample sizes in the future.

### 4.2 Emergence times versus active daylength

Initially, using the data from the egg laying phase, we found a positive relationship between emergence time and clutch initiation date, which is in accordance with results of a recent study on great tits and dark eyed junco's (*Junco hyemalis*, Graham et al., 2017). However, this relationship had not been found in captive great tits (Helm & Visser, 2010), in free‐living European starlings (Maury et al., 2020) and in free‐living blue tits (awakening time was used, which is highly correlated with emergence time, Steinmeyer et al., 2013). One possible explanation for the discrepancy between these studies might be that environmental factors that could affect the relationship may vary from year to year. For example, spring temperature may modulate the effect of light as trigger for the onset of breeding (Dominoni et al., 2020). Studying the relationship between chronotype and clutch initiation date in multiple years may reveal the impact of such environmental variation. As we hypothesized above, another possibility could be that active daylength rather than emergence time plays a role in determining onset of egg laying. Our initial analyses indeed show that individuals with longer active daylengths initiated egg laying earlier in the season and that individuals that emerged earlier from the nest box entered it later compared with late rising individuals (Maury et al., 2020, Steinmeyer et al., 2010, but Stuber et al., 2015 only showed this effect within individuals).

However, emergence time, entry time, and active daylength were measured during egg laying, and as a consequence, they were measured soon after clutch initiation (i.e., most often, measurements started 1 or 2 days after clutch initiation). The date of measurement was thus very tightly linked to the laying date of the first egg and differences in activity patterns between early and late laying females could possibly be explained by environmental changes over time (e.g., temperature, food availability, predation risk, and light intensity at the nest box due to an increase in leaf coverage) instead of intrinsic differences in chronotypes. Therefore, we expected that the date of measurement may be a confounding factor when analyzing the relationships between the activity parameters and clutch initiation date.

To tackle this problem, and because we did not find repeatability in the daily timing of activity between the different periods, we performed additional analyses that supported our presumption that the date of measurement is a confounding factor in the relationship between activity patterns and clutch initiation date. First, we tried to statistically correct for date of measurement by using individual intercepts and slopes. However, it is not possible to disentangle the date of measurement from clutch initiation date with this method. Instead, we used emergence times and active daylengths from a subset of females, that were measured during the pre‐egg laying phase. All females were measured multiple times within a range of 5 days (i.e., the date of measurement was independent from the clutch initiation date). We found that neither emergence time nor active daylength measured during the pre‐egg laying phase were related to the initiation of egg laying. Thus, as emergence times and active daylengths were not related to clutch initiation date when methodologically corrected for the date of measurement, we consider it most likely that the relationships we initially found are confounded by the date of measurement.

### 4.3 Variation in emergence time and active daylength across the breeding season

The date on which an individual was measured affected its emergence time, entry time, and active daylength relative to sunrise and sunset, that is, even after correcting for changes in sunrise and sunset over time. Emergence time delayed with date, while entry time advanced. A similar effect was recently reported in individual blue tits during the egg laying phase, but date effects on the population level were not investigated (Schlicht & Kempenaers, 2020).

As circadian clocks are entrained by the light–dark cycle (e.g., Berson et al., 2002; Wright Jr et al., 2013; Zeng et al., 1996), light intensity is likely a very important determinant for activity patterns in the wild (see also Sockman & Hurlbert, 2020 for a discussion on the role of active daylength on migratory behavior). In great tits and blue tits, light intensity at the nest box significantly influenced emergence time and awakening time in the morning, respectively (Steinmeyer et al., 2010; Stuber et al., 2015). However, the variation that we observed in emergence and entry time relative to sunrise and sunset over time both within and among individuals, indicates that the light intensities that trigger emergence from and entry into the nest box change over time.

At present, we can only speculate about the underlying drivers. During winter, when the days are short, individuals may have to make use of the full daylight period, while in spring, when the days are much longer, they may not need the full daylight period to perform all necessary tasks. Conversely, great tits may also need a minimal amount of sleep. Therefore, emergence times may delay relative to sunrise when the days lengthen while entry times advance. However, during the breeding season, we would then expect the absolute active daylength to remain constant from a certain moment onwards, but in fact, it started to decrease while the daylight period was still lengthening.

In addition to light intensity alternative zeitgebers (i.e., environmental factors that can entrain the biological clock) and masking factors (i.e. factors that do not change the internal clock time, but instead modify the expression of behavioral rhythms) may be important (Helm et al., 2017). For example, earlier studies showed that wild great tits delayed entry times on warmer evenings (Stuber et al., 2015), while captive great tits had later activity onset and earlier activity offset in warmer conditions (Lehmann et al., 2012). We found that an increase in maximum temperature was related to longer active daylengths, although temperature at sunrise and sunset did not significantly influence emergence and entry times. Temperature may thus modulate the activity patterns, but it could not fully explain the changes over time as observed in our population.

Another environmental factor that could affect emergence and entry time is the food availability (Hau & Gwinner, 1997, Rani et al., 2009, Vivanco et al., 2010). When food is not continuously available, but only during specific time frames, this can entrain the biological clock and individuals may shift their circadian phase, to meet the requirements of optimal foraging. Alternatively, food availability may have acted as a masking factor. For example, on days with high food availability great tits may need less time for foraging in order to meet their energy requirements, which enables earlier cessation of activity in the evening (Bach et al., 2017, Northeast et al., 2020, but see Inoue et al., 2016). However, as we do not have data on food availability, the influence of environmental factors like food availability on emergence and entry times needs further investigation.

Furthermore, the amount of time spent on night time incubation may have affected activity patterns during egg laying. During this phase, females already start incubating the eggs at night. With each subsequent egg, the amount of time spent on night time incubation increases (Lord et al., 2011; Podlas & Richner, 2013) and females with late egg laying dates incubate longer at night than females with early laying dates (Haftorn, 1981). Night time incubation normally starts immediately after entering the nest box, but whether entry times advance when night time incubation increases is yet unknown. Yet, none of the above‐mentioned factors seems to fully explain the observed temporal patterns in emergence and entry times.

As pointed out above, the significance of sunrise and sunset for determining activity patterns changes over time both within and among individuals. This is relevant for interpreting this and previous studies, even though most of the previous studies did not find date effects on emergence times during the breeding season (Graham et al., 2017; Maury et al., 2020; Womack, 2020). This discrepancy may be caused by our much larger sample size and a larger range of dates on which we measured emergence times. Therefore, it is possible that although Graham et al. (2017) did not find date effects on emergence times, their results may still be confounded by date. They measured emergence times during the incubation period, which is slightly different from our approach. However, if emergence time is measured at a fixed time after clutch completion, it could still be possible that date inflates the relationship between clutch initiation date and emergence time. In fact, Steinmeyer et al. (2013), who recorded sleep behavior during multiple months in winter in multiple years, found that sleep parameters (including awakening time) varied greatly between recording dates and therefore they corrected awakening times for the date of measurement. The corrected awakening times then again did not affect clutch initiation dates.

## 5 CONCLUSIONS

We showed that both emergence time and active daylength (measured during the egg laying phase) were related to clutch initiation date, but both relationships were confounded by date of measurement, as the timing of measuring activity patterns was tightly coupled to the initiation of egg laying. When using methodologically corrected data from the pre‐egg laying phase, we did not find a significant relationship between timing of activity and clutch initiation date. Furthermore, our results showed that the relevance of sunrise and sunset for the timing of activities varies throughout the breeding season, possibly in response to environmental factors, such as temperature or food availability. This makes it methodologically extremely challenging to correct for date of measurement effects. Future studies on functional consequences of activity patterns should hence aim to vary the time span between the dependent (here: laying date) and independent (here: timing of activity) variable, for example, by measuring activity patterns of all individuals on the same day(s), while being in the same breeding phase. Such confounding factors are possibly very common in statistical analyses including date. In addition, if individuals respond plastically to temporal changes in the environment, spatial differences in the environment may also affect activity patterns and could be partially responsible for differences in emergence times, entry times, and active daylengths among individuals, which as yet needs to be investigated.

## AUTHOR CONTRIBUTIONS

**Marjolein Meijdam:** Conceptualization (equal); formal analysis (equal); investigation (equal); methodology (equal); writing – original draft (equal). **Wendt Müller:** Conceptualization (equal); methodology (equal); writing – review and editing (equal). **Bert Thys:** Formal analysis (equal); writing – review and editing (equal). **Marcel Eens:** Conceptualization (equal); methodology (equal); writing – review and editing (equal).

## CONFLICT OF INTEREST

The authors declare no conflict of interest.

## Supporting information


Figure S1

Figure S2

Appendix S1

## Data Availability

All data that support the findings of this study are available via Dryad (https://doi.org/10.5061/dryad.2rbnzs7rk).
