# Supplementary figures and images for: No relationship between chronotype and timing of breeding when variation in daily activity patterns across the breeding season is taken into account
Source: Ecol Evol. 2022 Sep 20;12(9):e9353. doi: 10.1002/ece3.9353 (PMC9490139; doi:10.1002/ece3.9353)

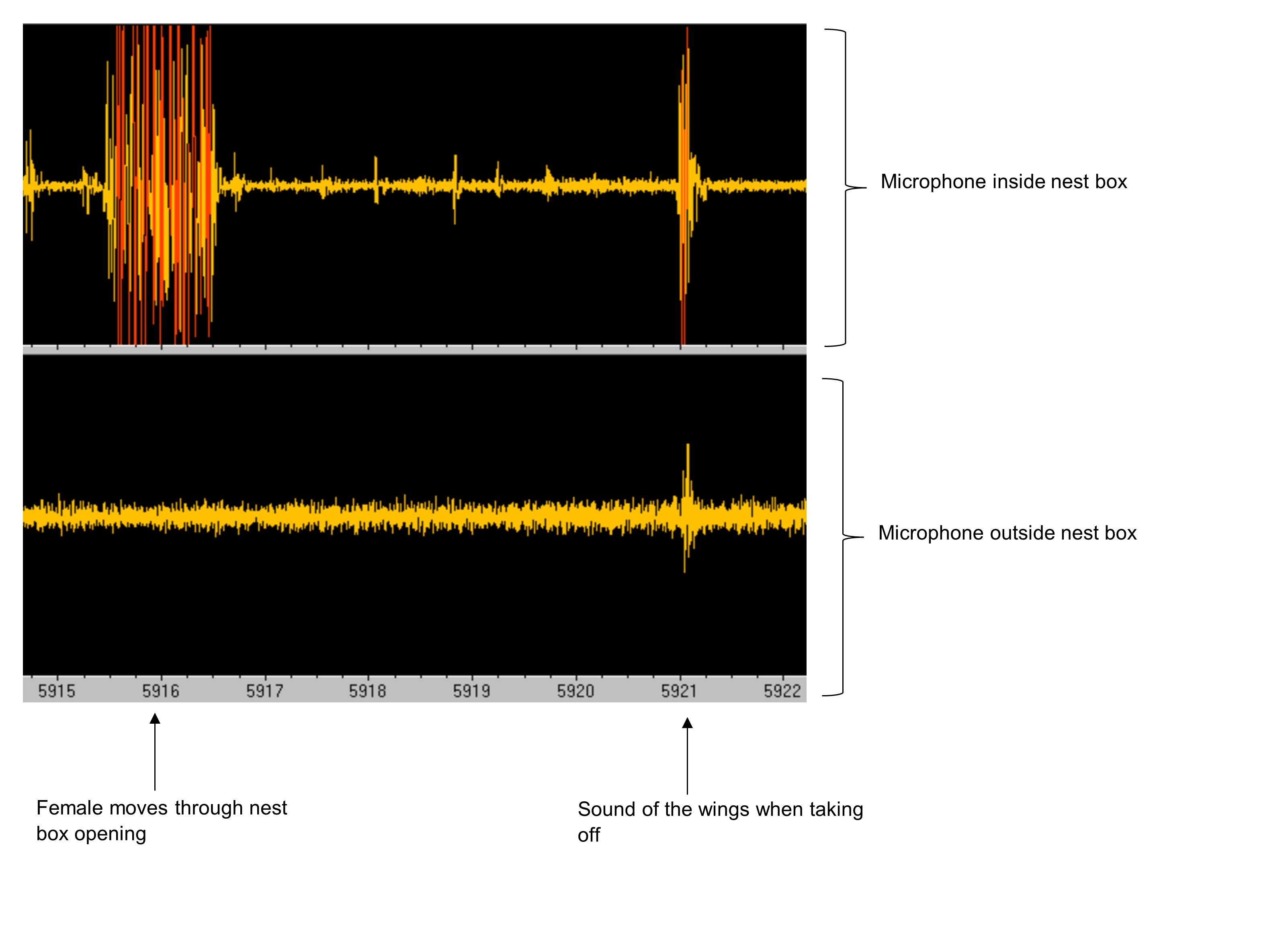

Supplement: Supplementary file 1 — Figure S1 [file ECE3-12-e9353-s003.png]

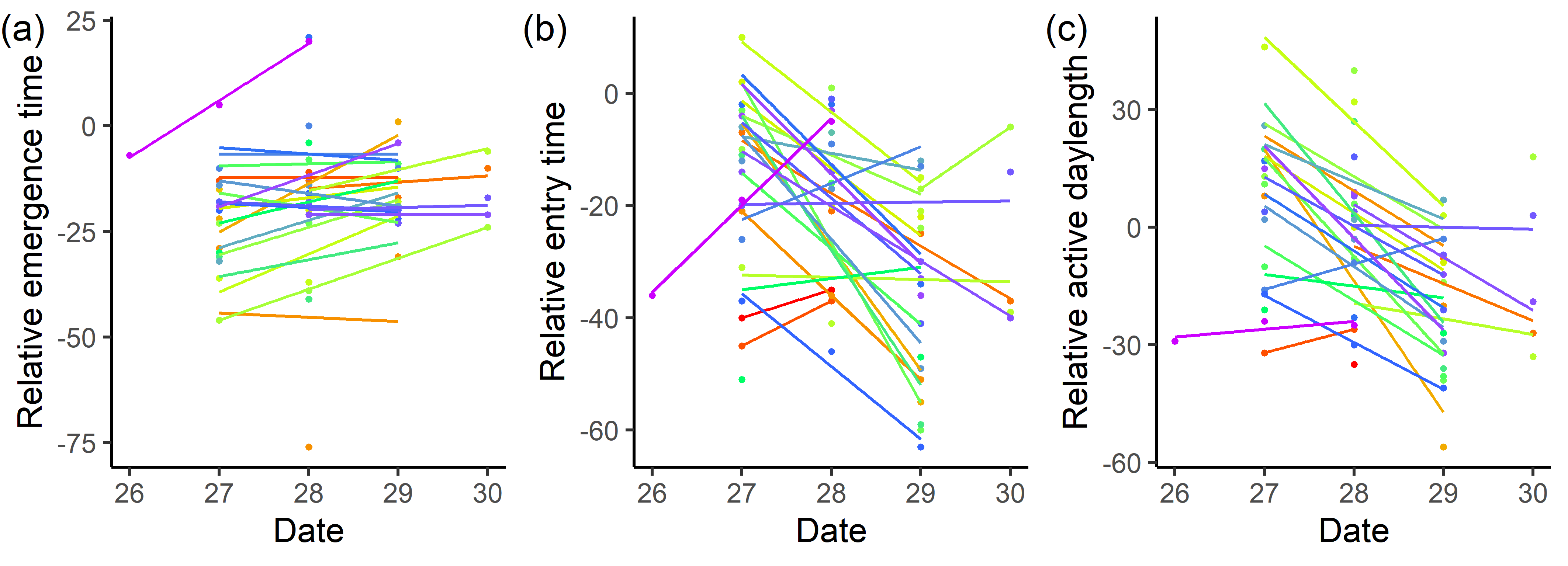

Supplement: Supplementary file 2 — Figure S2 [file ECE3-12-e9353-s002.tiff]
