# Supplementary material for: No relationship between chronotype and timing of breeding when variation in daily activity patterns across the breeding season is taken into account
Source: Ecol Evol. 2022 Sep 20;12(9):e9353. doi: 10.1002/ece3.9353 (PMC9490139; doi:10.1002/ece3.9353)
Supplement: Supplementary file 3 — Appendix S1 [file ECE3-12-e9353-s001.docx]

**Appendix table**

Table A1: Number of measurements per day relative to clutch initiation during the pre-egg laying and the egg laying phase.

| **Pre-egg laying phase** | | | | | | | | | | | | | | | | | | |
| --- | --- | --- | --- | --- | --- | --- | --- | --- | --- | --- | --- | --- | --- | --- | --- | --- | --- | --- |
|  | Number of days prior to clutch initiation | | | | | | | | | | | | | | | | | |
|  | -17 | -16 | -15 | -14 | -13 | -12 | -11 | -10 | -9 | -8 | -7 | -6 | -5 | -4 | -3 | -2 | -1 |  |
| Emergence time | 1 | 3 | 3 | 3 | 3 | 2 | 3 | 5 | 6 | 6 | 4 | 5 | 3 | 6 | 6 | 6 | 3 |  |
| Entry time | 2 | 2 | 3 | 4 | 3 | 2 | 3 | 5 | 7 | 6 | 5 | 4 | 4 | 6 | 6 | 7 | 4 |  |
| Active daylength | 1 | 2 | 3 | 3 | 2 | 2 | 3 | 5 | 6 | 6 | 4 | 4 | 3 | 6 | 6 | 6 | 3 |  |
| **Egg laying phase** | | | | | | | | | | | | | | | | | |  |
|  | Number of days after clutch initiation | | | | | | | | | | | | | | | | |  |
|  | 0 | 1 | 2 | 3 | 4 | 5 | 6 | 7 |  |  |  |  |  |  |  |  |  |  |
| Emergence time | 62 | 94 | 102 | 87 | 74 | 39 | 4 | 3 |  |  |  |  |  |  |  |  |  |  |
| Entry time | 93 | 101 | 85 | 76 | 45 | 9 | 2 | 0 |  |  |  |  |  |  |  |  |  |  |
| Active daylength | 55 | 89 | 73 | 74 | 39 | 8 | 2 | 0 |  |  |  |  |  |  |  |  |  |  |

Table A2: Results from linear mixed effects models for testing the influence of date on emergence time, entry time and active daylength (in minutes relative to sunrise, sunset and the period between sunrise and sunset respectively) during the pre-egg laying phase. Estimates in bold are statistically significant (P < 0.05).

| Dependent variable | *Fixed effects* | β | SE | t | df | *P* |
| --- | --- | --- | --- | --- | --- | --- |
| Emergence time | Date | 2.24 | 1.61 | 1.39 | 53.52 | 0.17 |
|  | Days prior to egg laying | -0.33 | 0.57 | -0.57 | 17.63 | 0.57 |
|  | Age | -4.72 | 3.63 | -1.30 | 17.11 | 0.21 |
|  | T_sunrise_ | -0.02 | 0.78 | -0.02 | 42.36 | 0.98 |
| Entry time | Date | 3.01 | 4.16 | 0.72 | 53.97 | 0.47 |
|  | Days prior to egg laying | 0.89 | 0.56 | 1.59 | 18.89 | 0.13 |
|  | Age | 0.60 | 3.64 | 0.16 | 17.89 | 0.87 |
|  | T_sunset_ | **3.50** | **1.28** | **2.73** | **48.61** | **<0.01** |
| Active daylength | Date | -0.65 | 3.58 | -0.18 | 46.14 | 0.86 |
|  | Days prior to egg laying | 0.69 | 0.86 | 0.80 | 16.29 | 0.44 |
|  | Age | 5.10 | 5.26 | 0.97 | 16.20 | 0.35 |
|  | T_max_ | **4.35** | **1.08** | **4.02** | **38.04** | **<0.001** |
